# Comparison of EML4-ALK fusion gene positive rate in different detection methods and samples of non-small cell lung cancer

**DOI:** 10.7150/jca.36580

**Published:** 2020-01-14

**Authors:** Shan Lu, Can Lu, YuXuan Xiao, Wei Zhu, QiuYan He, Bin Xie, JianHua Zhou, YongGuang Tao, Shuang Liu, DeSheng Xiao

**Affiliations:** 1 Department of Pathology, Xiangya Hospital, Central South University, Changsha, Hunan 410078 China; 2 Department of Pathology, School of Basic Medicine, Central South University, Changsha, Hunan 410078 China; 3 Hengyang medical college, university of south China, Hengyang, Hunan 421001 China; 4 Cancer Research Institute, School of Basic Medicine, Central South University, Changsha, Hunan, 410078 China; 5 Key Laboratory of Carcinogenesis and Cancer Invasion (Central South University), Ministry of Education, Hunan, 410078 China; 6 Key Laboratory of Carcinogenesis (Central South University), Ministry of Health, Hunan, 410078 China; 7 Department of Oncology, Institute of Medical Sciences, Xiangya Hospital, Central South University, Changsha, Hunan, 410008 China

**Keywords:** EML4-ALK fusion gene, immunohistochemistry, reverse transcription-polymerase chain reaction, next-generation sequencing, non-small cell lung cancer

## Abstract

**Objective**: To evaluate differences of EML4-ALK positive rates in tissues samples between immunohistochemistry, reverse transcriptase polymerase chain reaction and the next-generation sequencing method. Besides, to compare the differences of EML4-ALK positive rates in blood samples and tissue samples by next-generation sequencing. The results provide a basis for the selection of a suitable EML4-ALK fusion gene detection method.

**Methods**: Immunohistochemistry analysis of EML4-ALK in tumors was performed on samples from 2631 patients with non-small cell lung cancer. The mutation of EML4-ALK in the tissue samples of 399 patients with non-small cell lung cancer was detected by reverse transcription polymerase chain reaction. Next-generation sequencing was used to detect the mutation of EML4-ALK in 1505 non-small cell lung cancer patients, including 1208 tissue samples and 297 blood samples.

**Results**: The positive incidence of EML4-ALK by immunohistochemistry was 7.11% (187/2631). Histologically, 9.51% (170/1787) of the samples were lung adenocarcinomas, and 2.01% (17/844) were squamous cell carcinomas. The positive rate of EML4-ALK was 8.52% (34/399) in 399 patients with non-small cell lung cancer, as detected by reverse transcription polymerase chain reaction; the mutation rate of adenocarcinoma was 11.62% (33/284), and the mutation rate of squamous cell carcinoma was 0.86% (1/115). In 1208 patients with non-small cell lung cancer with tissue samples, the positive rate of EML4-ALK was 4.88% (59/1208), as determined by next-generation sequencing, the mutation rate of adenocarcinoma was 5.84% (58/994), and the mutation rate of squamous cell carcinoma was 0.47% (1/214). The positive rate of EML4-ALK detected by reverse transcription polymerase chain reaction was higher than that detected by immunohistochemistry. Compared with the next-generation sequencing results, the positive rates of EML4-ALK detected by immunohistochemistry and reverse transcription polymerase chain reaction were higher, and the differences were significant (p<0.05). In blood samples from 297 patients with non-small cell lung cancer, the positive rate of EML4-ALK detected by next-generation sequencing was 3.70% (11/297), the mutation rate of adenocarcinoma was 3.82% (10/262), and the mutation rate of squamous cell carcinoma was 2.86% (1/35). The EML4-ALK positive rate of the tissue samples was thus higher than that of the blood biopsy samples.

**Conclusion**: Among the three methods for detecting EML4-ALK, reverse transcription polymerase chain reaction has the highest positive rate, followed by immunohistochemistry, and next-generation sequencing has the lowest positive rate. The positive detection rate of EML4-ALK in tissue samples by next-generation sequencing was higher than that in blood samples.

## Introduction

Lung cancer has among the highest morbidity and mortality of all cancer types, and it is responsible for the highest rate of cancer-related mortality in both males and females 1. Primary lung cancer is mainly divided into two pathological types: small cell lung cancer (SCLC) and non-small cell lung cancer (NSCLC), of which NSCLC accounts for approximately 85% of lung cancer cases, mainly including adenocarcinoma, squamous cell cancer and other subtypes 2. Treatment methods for lung cancer mainly include surgical resection, chemotherapy and molecular targeted therapy 3. The main reasons for the high mortality rate of lung cancer are as follows: first, the onset of lung cancer is insidious and difficult to detect at an early stage, and 70% of the patients are in the middle or late stage at the time of diagnosis. Second, advanced lung cancer has poor sensitivity to conventional chemotherapy and poor prognosis. Therefore, early diagnosis of lung cancer is crucial to improving the survival rate of lung cancer. In recent years, with the rapid development of molecular biology, lung cancer driver genes have been continuously found and confirmed, promoting the emergence of corresponding molecular targeted drugs and entering the era of targeted drug therapy.

After the first drug target, the epidermal receptor factor EGFR, was discovered in NSCLC 4, Soda et al. 5 found the echinoderm microtubule-associated protein-like 4 anaplastic lymphoma kinase (EML4-ALK) fusion gene, which can induce the occurrence of lung cancer, in lung adenocarcinoma patients in 2007. Previous studies have found that EML4-ALK fusion is mutually exclusive with other carcinogenic factors, such as EGFR, ROS1, KRAS and other genes 6. Therefore, detection of the eml4-alk fusion gene is of great significance for targeted therapy 7. Timely target definition and timely treatment with a tyrosine kinase inhibitor (TKI) can play a crucial role in improving the survival and prognosis of patients 8, 9.

Currently, common clinical methods for the detection of EML4-ALK include immunohistochemistry (IHC), fluorescence in situ hybridization (FISH), reverse transcription polymerase chain reaction (RT-PCR) and next-generation sequencing (NGS) 10-15. In recent years, blood biopsy has become a hot spot of research due to its simple acquisition, small trauma and high repeatability. Blood samples are becoming an important source of samples for genetic testing 16-18, but whether blood samples can replace tissue samples for genetic testing is still controversial.

In our study, IHC, RT-PCR and NGS were used to detect the EML4-ALK fusion gene in tissue samples from NSCLC patients. NGS was used to detect EML4-ALK fusion gene mutations in tissue samples and blood samples of NSCLC patients. This study mainly explored the difference in the positive rate of the EML4-ALK fusion gene detected by different methods and in different samples, and this study provided a basis for the selection of methods for detecting driver genes in clinical NSCLC patients.

## Materials and Methods

### Ethics, consent and permissions

Approval to review, analyze, and publish the data in this study was given by the Ethics Board of Xiangya Hospital of Central South University. Written informed consent for the collection of medical information was obtained from all patients at their first visit.

### Clinical data

We collected 2631 primary NSCLC samples at the Xiangya Hospital of Central South University between January 2015 and December 2018 for VENTANA EML4-ALK (D5F3) immunohistochemical detection. These samples included 1787 adenocarcinomas and 844 squamous cell carcinomas. The samples included biopsies collected using CT-guided percutaneous lung puncture, bronchoscopy tissues, samples of exfoliated cell pellets from pleural effusions, and lymph node-puncture tissues. All samples were obtained before treatment, fixed with 4% neutral formaldehyde, embedded in paraffin, sectioned and stained with HE; the senior pathologist read the slides to make a clear diagnosis of NSCLC and determined that there were enough tumor cells for subsequent molecular pathological examination.

We collected 399 primary NSCLC samples from the Xiangya Hospital of Central South University between January 2017 and December 2018 for the detection of the EML4-ALK fusion gene. These samples included 284 adenocarcinomas and 115 squamous cell carcinomas. The samples included biopsies collected using CT-guided percutaneous lung puncture, bronchoscopy tissues, samples of exfoliated cell pellets from pleural effusions, and lymph node-puncture tissues. All samples were obtained before treatment, fixed with 4% neutral formaldehyde, embedded in paraffin, sectioned and stained with HE; the senior pathologist read the slides to make a clear diagnosis of NSCLC and determined that there were enough tumor cells for subsequent molecular pathological examination.

We collected 1505 primary NSCLC samples from the Xiangya Hospital of Central South University between January 2016 and December 2018 for the detection of the EML4-ALK fusion gene by NGS, including 1208 tissue samples and 297 blood samples. These tissue samples included 994 adenocarcinomas and 214 squamous cell carcinomas. The median age of the patients was 59 years (ranging from 29 to 89 years). Among the patients, 701 were males, accounting for 58%, and 507 were females, accounting for 42%. The samples included biopsies collected using CT-guided percutaneous lung puncture, bronchoscopy tissues, samples of exfoliated cell pellets from pleural effusions, and lymph node-puncture tissues. All samples were obtained before treatment, fixed with 4% neutral formaldehyde, embedded in paraffin, sectioned and stained with HE; the senior pathologist read the slides to make a clear diagnosis of NSCLC and determined that there were enough tumor cells for subsequent molecular pathological examination. Blood samples of 297 NSCLC patients were collected, among which 55% (163/297) were male and 45% (134/297) were female. According to histological classification, there were 262 cases of adenocarcinoma and 35 cases of squamous cell carcinoma.

### Reagents

The anti-EML4-ALK (D5F3) rabbit monoclonal antibody, Ventana OptiView signal amplification kit, OptiView IHC detection kits, rabbit monoclonal negative quality control antibody and quality control slice were obtained from Roche Diagnostics Product Co. LTD, Tucson, U.S.A.

A DNA extraction kit (FFPE DNA, Cat NO.ADx-TI01), a DNA/RNA extraction kit (FFPE DNA/RNA) and a human multigene mutation detection kit (PCR fluorescence probe method) were purchased from Amoy Diagnostics Co Ltd, Xiamen, China.

A human multigene mutation detection kit was purchased from Burning Rock Biotech, Guangzhou, People's Republic of China; a human multigene mutation detection capture probe (Lung cure ™ LK101, Lung-cure™ LK165 (Blood Version)) was purchased from Burning Rock Biotech, Guangzhou, People's Republic of China.

### VENTANA immunohistochemical analysis

After 3 mm thick paraffin slices were baked, the slices were dyed directly using the Benchmark XT automatic immunohistochemical dyeing machine (American VENTANA company). The dyeing procedure was performed according to the instructions provided with the kit. The test results were evaluated using light microscopy. A binary method of interpretation was used as follows: strong granular cytoplasmic staining (any percentage) in the tumor cells was scored EML4-ALK (+); otherwise, they were scored EML4-ALK (-).

### DNA extraction

A total of 10 pieces that were 5-8 microns thick were sectioned from wax blocks selected from those with more tumor tissues, and 75% alcohol was used to sterilize the equipment between each sample to prevent cross-contamination. The tumor tissue genomic DNA was extracted using an AmoyDx DNA extraction kit. After the extraction of the DNA, its concentration and quality were evaluated using a Nanodrop ultramicro spectrophotometer, with a quality criterion of 1.7 < OD260 / OD280 < 2.1.

### Amplification-refractory mutation system (ARMS) analysis

Mutational analysis of the EML4-ALK was carried out according to the ARMS method using a human multigene mutation detection kit (PCR fluorescence probe method) from Amoy Diagnostics Co Ltd, Xiamen, China. We detected 26 fusion gene mutations of the ALK gene using this procedure. These included 22 EML4-ALK fusion gene mutations and 4 other ALK fusion gene mutations. The EML4-ALK fusion gene mutations were detected using CFX96-type fluorescent quantitative PCR from Bio-Rad company. Each batch of reactions was set up to include simultaneous positive and negative controls. After the reaction, the fluorescent signal curves and the threshold line were used to interpret the mutation results.

### NGS analysis

DNA was extracted from paraffin-embedded tissue samples of patients using a nucleic acid extraction kit (Model: FFPE DNA, Cat NO. ADx-TI01, Xiamen Aide Biomedical Technology Co., Ltd.), and the concentration of DNA was determined using the Qubit dsDNA Assay. The genomic DNA was disrupted using the covaris M220 ultrasound system. The universal kit for multigene mutation detection (Guangzhou Burning Stone Medical Laboratory Co., Ltd., item number: RS03F-12) was used for end repair, 3' end plus A, linker ligation, PCR amplification, and purification of the magnetic beads to obtain a pre-library. Then, we used the human multigene mutation detection capture probe (Lung cure ™ LK101, Lung-cure™ LK165 (Blood Version), Guangzhou Burning Stone Medical Laboratory Co., Ltd.) hybrid to capture the target area, with streptomycin affinity magnetic beads capturing the library fragments that hybridized with the probe, and we used PCR for amplification and purification to determine the size and concentration of the library fragment. The libraries were mixed, denatured, and sequenced on a genetic sequencer (Illumina MiSeq Dx) platform. After the completion of the sequencing, the sequence was analyzed and compared with the second-generation sequencing data analysis software (produced by Guangzhou Burning Stone Medical Laboratory Co., Ltd.), and the results were interpreted.

### Sequence data analysis

Sequence data were mapped to the human genome (hg19) using BWA aligner 0.7.10. PCR duplicate reads were removed before base substitution detection. Local alignment optimization and variant calling and annotation were performed using GATK 3.2. DNA translocation analysis was performed using both Tophat2 and Factera 1.4.3.

### Statistical analysis

The chi-square test (or Fisher's exact test) and independent samples test were applied to explore the univariate association between the clinic pathological variables and the specific genetic aberrations for the categorical and continuous data for EGFR and EML4-EML4-ALK. All statistical calculations were performed using SPSS version 19.0 (SPSS, Inc., Chicago, IL). A two-tailed P value of 0.05 was considered significant.

## Results

### The comparison of EML4-ALK positive rate in tissue samples among IHC, RT-PCR and NGS

**Figure 1:**
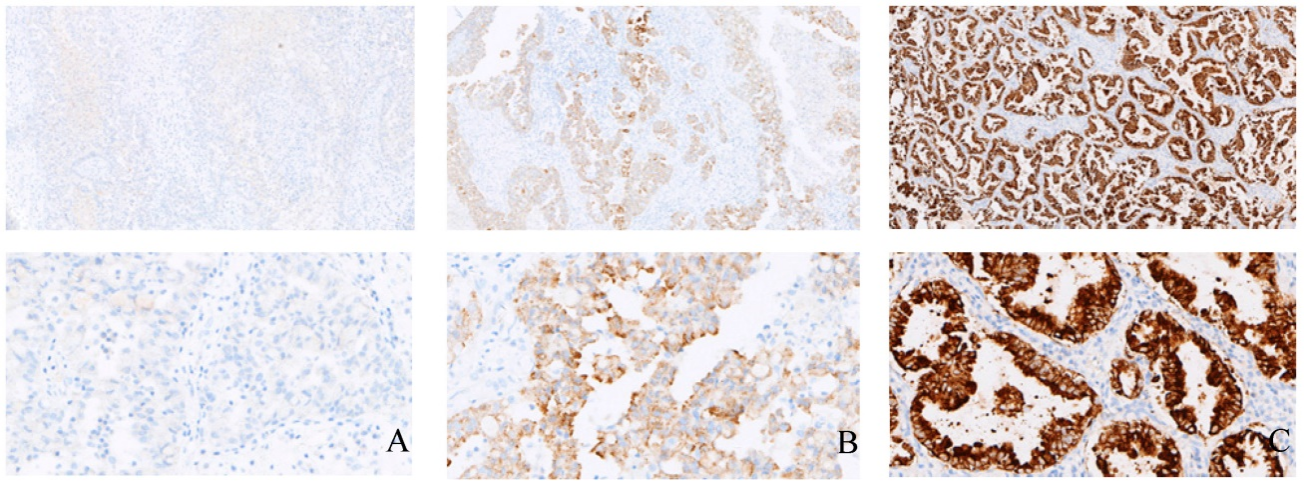
Examples of the immunohistochemical analysis of EML4-ALK **in tissue samples**. A, B, and C show the negative control, positive control and an EML4-ALK (+) case (strong granular cytoplasmic staining) (×100), respectively; D, E, and F show the negative control, positive control and an EML4-ALK (+) case (strong granular cytoplasmic staining) (×400), respectively.

**Figure 2:**
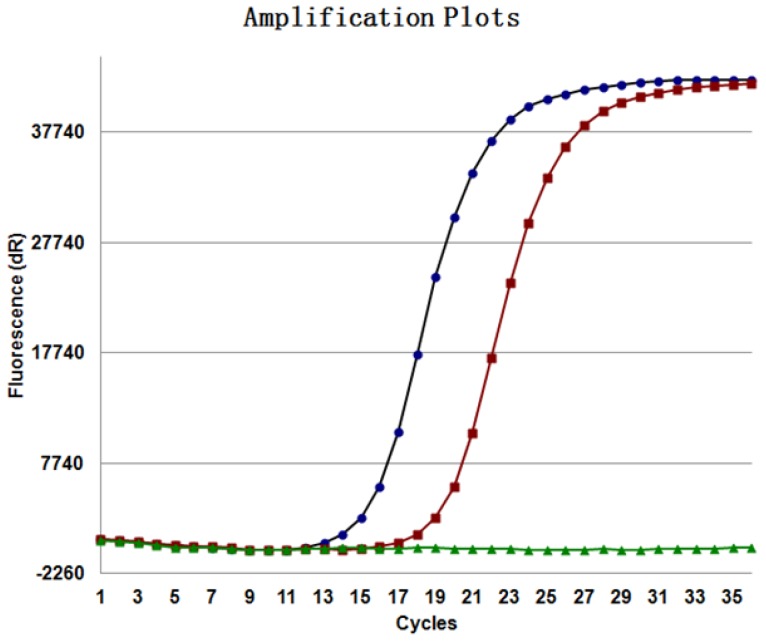
EML4-ALK **in tissue samples** were detected by RT-PCR (ARMS method).

IHC was used to detect the expression of EML4-ALK in tissue samples of 2631 patients with NSCLC; 187 cases were positive (Figure 1), the positive rate was 7.11% (187/2631), and the positive rate in adenocarcinoma was 9.51% (170/1787) and 2.01% (17/844) in squamous cell carcinoma. The EML4-ALK fusion gene was detected by RT-PCR in samples from 399 NSCLC patients. Among them, EML4-ALK fusion gene was detected in 34 patients (Figure 2), the positive rate was 8.52% (34/399), the positive rate in adenocarcinoma was 11.62% (33/284), and the positive rate in squamous cell carcinoma was 0.86% (1/115). The NGS method was used to detect the EML4-ALK fusion gene in tissue samples of 1208 patients with NSCLC. Fifty-nine EML4-ALK-positive cases were identified, and the positive rate was 4.88% (59/1208); the positive rate was 5.84% (58/994) in adenocarcinoma and 0.47% (1/214) in squamous cell carcinoma. Among the above three detection methods, the positive rate of RT-PCR was the highest, followed by IHC, and that of the NGS method was the lowest. There was no significant difference between the IHC and RT-PCR method groups (p>0.05). IHC and RT-PCR were compared with NGS, and the between-group difference in EML4-ALK positive rate was statistically significant (p<0.05) (Table 1). The detection of EML4-ALK by these three methods was significantly higher in patients with adenocarcinoma than in patients with squamous cell carcinoma.

### The comparison of EML4-ALK positive rate between tissue and blood samples

The NGS method was used to detect the EML4-ALK fusion gene in 297 blood samples. Eleven cases of EML4-ALK were scored positive; the positive rate was 3.70%, and the positive rate in adenocarcinoma was 3.82% (10/262). The positive rate in squamous cell carcinoma was 2.86% (1/35). The positive rate of tissue samples was higher than that of blood samples, but the between-group difference was not statistically significant (P>0.05) (Table 2). The positive rate of EML4-ALK in female tissue samples was 4.93% (25/507), and that in blood samples was 5.22% (7/134). The difference between them was not statistically significant. The positive rate of EML4-ALK in male patients in tissue samples was 4.85% (34/701) and in blood samples was 2.45% (4/163), and the difference between them was not statistically significant. In patients with adenocarcinoma, the positive rate of EML4-ALK in tissue samples was 5.84% (58/994), and that in blood samples was 3.82% (10/262). The difference between them was not statistically significant (P>0.05). In patients with squamous cell carcinoma, the positive rate of EML4-ALK in tissue samples was 0.47%, and that in blood samples was 2.86% (P<0.05) (Table 2).

## Discussion

Every year, more than 2 million people are diagnosed with lung cancer, and approximately 1.5 million people die of lung cancer. Although the mortality rate of lung cancer patients has decreased, the 5-year survival rate is still not optimistic, only approximately 18% 19. ALK rearrangements are caused by reversal or ectopic rearrangement on chromosome 2, and the anaplastic lymphoma kinase fusion gene is the most common type 20, 21. The EML4-ALK gene is located at 2p23.2, is 729 kb in length, and belongs to the type I transmembrane tyrosine kinase protein of the insulin superfamily 22, 23. EML4-ALK has carcinogenic and malignant transformation properties in vitro and in vivo 24, 25. Among all the fusion genes, EML4-ALK is the most common fusion gene type, generally 4-7% in both the Western and Chinese population, which is approximately consistent with our research results. IHC was used to detect the expression of EML4-ALK in tissue samples of 2631 patients with NSCLC, 187 cases were positive, the positive rate was 7.11%. The EML4-ALK fusion gene was detected by RT-PCR in samples from 399 NSCLC patients. Among them, EML4-ALK fusion gene was detected in 34 patients, the positive rate was 8.52%. The NGS method was used to detect the EML4-ALK fusion gene in tissue samples of 1208 patients with NSCLC. Fifty-nine EML4-ALK positive cases were identified, and the positive rate was 4.88%. It is common in patients who do not smoke or smoke less, the number of EML4-ALK patients in Asia is higher than in the West, and men account for 50-60% of cases 28. Since crizotinib has been approved by the FDA for the treatment of EML4-ALK-positive NSCLC patients, it has been clinically proven to greatly improve the prognosis of positive patients 29. Crizotinib can be targeted to EML4-ALK, ROS1, and MET for treatment. Gene detection is the premise of precise targeted therapy. Our study is based on a large number of NSCLC patients, using IHC, RT-PCR, and NGS methods for detection and comparison of the EML4-ALK gene, providing a basis for rational selection of clinical applications. The gold standard for predictable EML4-ALK detection is fluorescence in situ hybridization (FISH) 30. However, FISH detection has limitations, such as high cost, signal instability, and difficulty in scoring, which is not easy implemented for widespread used. Therefore, it is very important to find a method other than the FISH method to detect the EML4-ALK gene. The IHC method is convenient and rapid, and it can be used as an effective screening method for EML4-ALK, but the sensitivity is poor, and the type of EML4-ALK mutant gene cannot be clarified 31-35. RT-PCR for detecting EML4-ALK has high sensitivity and specificity, but it requires high-quality samples and can cause false-negative and false-positive results. NGS is a new technology used in clinical gene detection. It can detect multiple mutations of multiple genes in a single sample with high sensitivity and reproducibility, but it needs to be confirmed by comparative studies for clinical application 36. In fact, FISH, IHC or RT-PCR can be used as diagnostic techniques for ALK-positive lung cancer, which is recommended by the Diagnostic and Therapeutic Guidelines for Primary Lung Cancer of the Chinese Society of Clinical Oncology. According to the types of tissue samples, the characteristics of molecular detection methods and laboratory conditions, effective detection methods are reasonably adopted. When doubting the reliability of one technology, another technology can be considered for validation. This study showed that, among the three methods for detecting EML4-ALK, the reverse transcription polymerase chain reaction method had the highest positive rate, followed by immunohistochemistry, and the next-generation sequencing method had the lowest positive rate. In our experiments, IHC only detected the EML4-ALK fusion gene, while RT-PCR detected the three fusion genes KIF5B-ALK, TFG-ALK and KLC1-ALK, in addition to the EML4-ALK fusion gene. This may be the reason why the positive rate of RT-PCR is higher than the IHC method. Tumor tissue biopsy samples are the main sample types for clinical testing, including bronchoscopy, ultrasound bronchoscopy or percutaneous lung biopsy 37, 38. However, tissue biopsy has many limitations. The acquisition of tumor tissue is an invasive operation, and surgical procedures may cause serious complications. In addition, due to the heterogeneity of the tumor, the mutation between the metastatic tumor in the body and the tumor cells of the primary tumor may only be the same for one-third of the mutations 39. To overcome these difficulties, noninvasive blood biopsy by NGS to detect circulating tumor DNA (ctDNA) in the blood has become a source of samples for NSCLC patients 40, 41. Blood biopsy can dynamically track dynamic changes, and blood collection can be repeated for real-time monitoring of treatment response and prognosis 42. However, compared with the traditional IHC and RT-PCR methods, the NGS method requires a substantial amount of clinical verification. Our study found that NGS detection of EML4-ALK was more positive in tissue samples than in blood samples. In terms of sex, there were no statistically significant differences between tissue samples and blood samples. In terms of histological type, there was no significant difference in adenocarcinoma between tissue samples and blood samples, but this study observed that in squamous cell carcinoma, the detection rate of EML4-ALK in blood biopsy samples was higher than that of tissue biopsy samples (P<0.05). This may be due to the lower sample size of this study, and further research is needed to further expand the sample size. These results indicate that the use of tissue samples is still the preferred option for the current detection of EML4-ALK, but in non-small cell lung cancer patients who are unable to obtain tissue samples, blood samples can be used as a supplement to tissue samples.

## Figures and Tables

**Table 1:** The comparison of EML4-ALK positive rate **in tissue samples** among IHC, RT-PCR and NGS

Group		Total cases	Mutation cases	Mutation rates
IHC	Adenocarcinoma	1787	170	9.5%
	Squamous carcinoma	844	17	2%
RT-PCR	Adenocarcinoma	284	33	11.6%
	Squamous carcinoma	115	1	0.9%
NGS	Adenocarcinoma	994	58	5.8%
	Squamous carcinoma	214	1	0.5%

**Table 2:** The comparison of EML4-ALK positive rate between tissue and blood samples

Group		Total cases	Mutation cases	Mutation rates	P
Male	Tissue samples	701	34	4.9%	0.209
	Blood samples	163	4	2.5%	
Female	Tissue samples	507	25	4.9%	0.826
	Blood samples	134	7	5.2%	
Adenocarcinoma	Tissue samples	994	58	5.8%	0.222
	Blood samples	262	10	3.8%	
Squamous carcinoma	Tissue samples	214	1	0.5%	0.262
	Blood samples	35	1	2.9%	
